# Harvard Odontological Society

**Published:** 1894-03

**Authors:** 

**Affiliations:** Harvard Odontological Society


### HARVARD ODONTOLOGICAL SOCIETY.

A MONTHLY meeting of the Harvard Odontological Society was held Thursday evening, September 28, 1893, at Young's Hotel, Boston, Dr. Eddy, president, in the chair.

*President Eddy.*—We have so often in our practice met with people who are anxious concerning the poor character of their teeth that we have felt the need of some system of feeding or system of nourishing which we could prescribe to them for the purpose of improving the quality of their teeth, and thereby relieve all this anxiety. We are happy to-night in being honored with the presence of Professor R. W. Greenleaf, who will bring before us the subject of "Foods."

(For Professor Greenleaf's paper, see page 163.)

DISCUSSION.

Dr. Hitchcock stated that he had recently read an article describing the appearance of several cases of typhoid fever that could not at first be accounted for. After investigation it was found that the milk furnished by a certain milkman was responsible. He had been in the habit of washing his cans in water taken from a well which was near a deserted house. At some time previous there had been a case of typhoid fever in the house, and the water which came from the well was contaminated by the typhoid germs. He did not adulterate his milk, but simply washed his cans in the water, thereby producing a great number of cases of typhoid fever in Watertown and Nonantum.

I would like to ask Professor Greenleaf's opinion in regard to those foods which were put upon the market some time ago to be used by women during gestation, lactation, etc. They were made in a form similar to the chocolate caramel and were supposed to contain the phosphates, so that the bony structures would be well supplied during those periods. I have forgotten now who prepared them. My idea was that the bad results following the changes which the sugar underwent would offset the good received from the extra amount of phosphates taken.

*Professor Greenleaf.*—I am not prepared to say, not knowing to what food you refer; but it would be possible for changes in the sugar to counteract any good effect which might be hoped for from the phosphates.

*Dr. Hitchcock.*—What would be the effect on the child?

*Professor Greenleaf.*—As the child derives its nourishment from its mother, it is obvious that what affects the mother may affect the child.

*Dr. Werner.*—It has been my study for years how to counteract the evil tendencies that my profession brings to me, and it seems that some of these thoughts that the essayist has put before us in regard to our diet—particularly where he speaks of varying it, and of the bad effects of eating beef three times a day—are worthy of attention.

*Dr. Briggs.*—I would like to ask Professor Greenleaf whether, in the different amounts of the proteids and carbohydrates required, there should not be a difference in the maintenance of their relations in the man who gets a great deal of out-door exercise,—for example, the lumbermen, who are employed in cutting timber, and the army men that he has spoken of,—when compared with men of sedentary habits, like ourselves; in other words, if we do not need to have the line of the proteid substances approach nearer to the carbohydrates than they do?

*Professor Greenleaf.*—I think that is a very fair question, and it should be answered in the affirmative, as the processes of cooking affect somewhat the value of any article as food-substance. If meats are properly cooked, they go further in furnishing nutritive material. But the point is that the needs are for about that proportion of the different substances, and by the proper processes of cooking those amounts can be obtained from smaller quantities of foods.

*Dr. Briggs.*—I referred to the needs rather than to the processes of cooking. The question is, Should not the man who gets little or no exercise, and gets less oxygen than another man of the same age, take more of the proteids in proportion to the carbohydrates than a man whose occupation is out of doors, inasmuch as the oxygen he does get is appropriated by the carbohydrates, and the proteids are non-utilized? Is there not that danger to a man who takes little air and no exercise?

*Professor Greenleaf.*—It may be so to a certain extent; I cannot give you the exact data on that matter.

*Dr. Hitchcock.*—I would like to ask Professor Greenleaf how it is that savages can eat meat that is uncooked and sometimes perfectly putrid, while we are poisoned by ptomaines, whether our food is cooked or not? Is food more injurious when it is cooked?

*Professor Greenleaf.*—That is also a very difficult question to answer satisfactorily. To be able to eat food of the character you mention, one must suppose a stomach capable of digestion to an extent that we know nothing of, and their tolerance of poisons would naturally be so different from those of the people on whom the statistics were based that I do not think I can give you any information as to what would be injurious or what foods they would require.

*Dr. Hitchcock.*—But are not the ptomaines taken into the system just the same with the savage people as with civilized people?

*Professor Greenleaf.*—Possibly; but we do not know what may go on in the light of elimination. Where immense quantities of food are taken, and there are given digestive organs of unknown capacity, very likely the process of elimination goes on more rapidly than we have been calculating. The processes of elimination and powers of resistance are so different among the savages, and our knowledge of their dietaries is so limited, that we cannot say what would be injurious to them. But there is another feature to be taken into consideration. In the composition of some foods there are certain elements which, when they are developed, act as poisons to some persons,—in other words, some people have idiosyncrasies in regard to certain kinds of foods. Strawberries, which are regarded as so wholesome by the majority of people, are extremely poisonous to some, as are also shell-fish and various other kinds of fish. We do not know exactly what the form of poison is, but we do know that individuals differ in their susceptibility to the action of different kinds of foods.

*Dr. Briggs.*—As an illustration that the hardier classes cannot always endure everything, I will mention an epidemic that occurred in a lumber camp in Maine near where I go every summer. The majority of the men were stricken with typhoid fever, and it was found, after investigation, that they had drunk water from a running brook into which, some distance above, a wounded deer had fallen and decomposed. The peculiar feature of the case was that, as the excreta from the patients were properly destroyed, the fever still spread, and it was decided after the deer was found that the cause was due to the water running over that putrid meat. If we do not accept that theory, I suppose we would have to account for it in some way that the deer had the typhoid germ. Perhaps it was not a real typhoid. At all events, it goes to show that rugged men cannot stand drinking poisons any more than we can.

*Professor Greenleaf.*—The case Dr. Briggs speaks of is certainly a very curious one. The evidence is so strong that typhoid fever originates only from other typhoid cases that we do not feel justified in crediting it to any other source. The origin is oftentimes exceedingly obscure, and the question might be raised whether in this case the typhoid may not have been brought into the camp on clothing or in the milk-supplies, or other articles of food. And then it is possible this may have been a fever simulating typhoid. The difficulties of diagnosis may have led the physicians into the error of pronouncing it typhoid.

*Dr. Briggs.*—I do not know what the local physician knew about the subject, but it was claimed that the origin of the epidemic was in the water which passed over the deer, and it is just as reasonable to say that the same disease could be caused by putrefactive changes in the camp itself.

*Professor Greenleaf.*—We do not recognize that as a cause either. We suppose that all cases of true typhoid must originate from the typhoid germ itself. There is, however, another thing to be considered in the case of an epidemic, and that is, secondary infection,—that is, a person will be nursing a patient, as might have been the case with the hunters we have just been discussing. Suppose, for instance, one of them had imbibed the typhoid germ in some way, and the others in nursing him and attending him were secondarily affected by the handling of articles soiled by the typhoid germs. The number of ways in which secondary infection can result are very numerous,—cutting bread, for instance, handling articles of soiled clothing and bed linen, using the same spoons and vessels from which medicine has been given without their being properly disinfected. This secondary infection is an element that probably accounts for a greater number of cases than the original infection by drinking water and the like.

*Dr. Giblin.*—I would like to ask Professor Greenleaf a question which was suggested to my mind by the frequent occurrence of typhoid fever in people returning to the city from vacations in the country, and it would be a valuable thing to know what precautionary measures should be taken in regard to food, etc., if it were thought there was likely to be any danger from typhoid. Dr. Grant tells me he has heard of several cases of friends who have returned from Chicago and had an attack of typhoid fever. I think it would be a great service to our friends if we knew some precautionary measures which we could recommend to them.

*Professor Greenleaf.*—That opens up a very extended subject. I do not know that I am right in taking up so much of your time, for I know you have another paper to discuss this evening, but the answer to the question the gentleman asks is particularly fitting just at present on account of the scare we have recently had from the threatened invasion of cholera. Now, that disease—cholera—is understood to be one in which, among other things, an inflammation of the intestinal mucous membrane is produced by a certain germ; those germs give rise to ptomaines which are toxic, and thus, when these germs are taken in either by drinking water or a like way, they at once set up an irritation in the intestines and produce the various symptoms which are known as cholera. That is the theory of the disease. Now, if we keep that clearly in mind, and also the fact that the typhoid germ is in a general way very similar in its action to cholera, only slower, we can see what precautionary measures are necessary to prevent such diseases.

The cholera epidemic, which raged in Hamburg and other parts of Europe (and some idea of its extent can be gained when I say that in Russia alone were one hundred and ninety-nine thousand cases), is still fresh in our minds. In Hamburg it started in with great virulence, attacking some three or four thousand persons, and a noticeable feature of this epidemic was that the German soldiers, who were in the midst of the infection in Hamburg, escaped cholera. It was also noticed that the town of Altona, though but a short distance from Hamburg, practically a part of it, escaped with very few cases. Berlin also, though not many miles from the scene of the epidemic, with its million and a half of people, had comparatively few cases, only thirty-two. Now, the immunity of the inhabitants of Altona and of Berlin was chiefly by virtue of their water-supply, which had been purified by proper filtration, and the immunity of the garrison at Hamburg was due to their very carefully avoiding the polluted Elbe water, which was practically unfiltered at Hamburg, and by their drinking water from an artesian well instead.

If we think of such places as our people visit in the summer, you can see how easy it is for them to become infected with typhoid. Some one will be ill with typhoid, and in caring for that person no attention whatever may have been paid to the disinfection of the excreta. It is the most important thing in taking care of such a person to have the discharges absolutely disinfected, but, as I say, attention is not always paid to it. We also know that in many instances in country places, excreta are deposited within a very short distance of the water-supply, and it is a very easy matter for wells near at hand to be contaminated, and the germs be thus taken in through the water. A few years ago in Nahant, where no typhoid had existed for years, some seventy cases arose in a few weeks' time. The history of those cases was traced to two or three imported ones whose discharges were not disinfected, hence the water-supply was contaminated, and all the other cases arose from them.

Now, if our families have been living near a house where there has been a case of typhoid fever, they may imbibe the germs in the same way, but it does not follow that every case of typhoid fever will spread a contagion, as many times where these precautions are not taken certain people escape the infection, which may be due to their respective conditions of health, which gives them immunity from the disease, or that the germ itself has in some way been destroyed. We cannot tell how persons escape the disease in such cases, but we do know that, given the danger of an exposure to a certain disease, results have proved the necessity of adopting all precautionary measures against it.

In Chicago the sewage material empties into Lake Michigan, and but a comparatively short distance from where the water-supply is obtained. Until recently there have practically been no provisions for the disinfection of this material. There is now in operation a system by which every particle that goes through the World's Fair grounds is disinfected; the solid portions are taken out, compressed, and burned, and the liquid waste containing disease-germs is thoroughly disinfected with chemicals, so that whatever sewage enters the lake at that point now is thoroughly purified. It will be a great advance to have all sewage similarly disinfected before it can enter a water-supply.

We were shocked when we heard of the great number of deaths from cholera that took place in Hamburg last year; but it is a fact not generally known, that the number of cases of typhoid in Chicago, Lowell, and Lawrence last year exceeded the proportion, when compared with the existing population, of the number of cases of cholera in Hamburg during the epidemic. You may set this down as a rule: that wherever you have a large number of cases diffused in a certain place, it is generally safe to say that the public water-supply is a possible source; where there are but a few isolated cases, the origin may be looked for in an infected milk-supply or other possible source less widely operating. If there is any reason to suspect that the source of a number of cases is in the water, the safest thing to do is to go without water, but in a great majority of instances that is a needless deprivation. A better way is to drink no water that has not been recently boiled if its purity is not above suspicion.

HENRY L. UPHAM, D.M.D.,
*Editor Harvard Odontological Society.*

